# Adaptive laboratory evolution of *Lactiplantibacillus plantarum* enhances furan tolerance and lactic acid production in lignocellulosic hydrolysate

**DOI:** 10.1186/s40643-026-01106-4

**Published:** 2026-08-03

**Authors:** Sharoni Sharma, Sarvesh V. Surve, Joyleen M. Fernandes, Vinit Mahale, Monty Vijayvargiya, Ram Kulkarni

**Affiliations:** Symbiosis School of Biological Sciences, Symbiosis International (Deemed University), Pune, 412115 India

**Keywords:** Adaptive laboratory evolution, Furfural, Furfuryl alcohol, 5-hydroxymethylfurfural (HMF), Lactic acid, *Lactiplantibacillus plantarum*, Lignocellulose

## Abstract

**Abstract:**

Furfural and 5-hydroxymethylfurfural (HMF) are two major lignocellulosic growth inhibitors that hinder microbial growth and fermentation of lignocellulosic hydrolysate for lactic acid production. In this study, we employed adaptive laboratory evolution (ALE) to enhance the tolerance of *Lactiplantibacillus plantarum* JGR2, a strain previously isolated in our lab, to furfural and HMF. The adapted strains demonstrated significantly improved growth in the presence of these inhibitors compared to the parental strains. Whole-genome resequencing revealed multiple mutations including high-impact non-conservative mutations in genes encoding DNA recombination and repair protein (RecF, lp_0005), flavin prenyltransferase (UbiX lp_0271), and oligo-1,6-glucosidase (lp_0189). Transcriptomic analysis indicated that adaptation elicited more pronounced differential gene expression compared to acute inhibitor exposure. Upon furfural exposure, the furfural-adapted isolate showed fewer differentially expressed genes than the parental strain, indicating a possible shift in the transcriptomic profile as a possible mechanism of furfural adaptation. Mechanistic investigation revealed that the adapted isolates reduce furfural into the less toxic furfuryl alcohol, suggesting a key detoxification mechanism. Notably, *lp_3051* (*dhaT*, 1,3-propanediol dehydrogenase) encoding furfural reductase activity was upregulated in both furfural- and HMF-adapted isolates. Membrane fatty acid analysis revealed increased unsaturated fatty acids and cyclopropane fatty acids in adapted strains. Finally, the adapted strains exhibited improved growth in rice straw hydrolysate and produced significantly higher relative lactic acid yields compared to the parental strain, thus demonstrating improved bioproduction under inhibitor-rich conditions. This study not only provides a comprehensive understanding of *L. plantarum*’s response to lignocellulosic inhibitors, but also yields evolved bacterial candidates for further scientific and industrial exploration.

**Graphical abstract:**

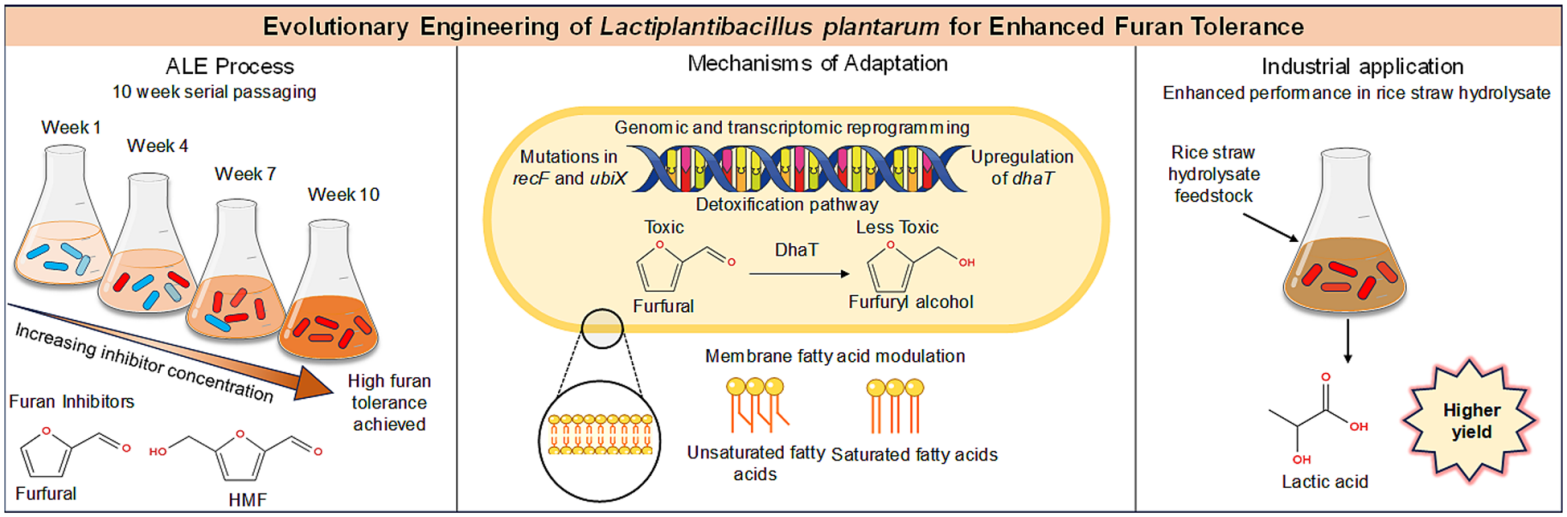

**Supplementary Information:**

The online version contains supplementary material available at 10.1186/s40643-026-01106-4.

## Introduction

Lignocellulose, a complex plant cell wall component, offers a feedstock for an ecological and economic route of producing valuable organic compounds through microbial fermentation. However, its polysaccharides are encased in lignin, requiring harsh pre-treatment to make them accessible to enzymes, such as cellulases, that can hydrolyse the polysaccharides, thus making them available for microbial fermentation. Pre-treatment methods, such as heat/steam explosion or acid hydrolysis, break down lignin but also generate inhibitory compounds such as furfural and 5-hydroxymethylfurfural (HMF), which hinder microbial growth and productivity (van der Pol et al. 2014).

Furfural and HMF inhibit microbial growth through multiple mechanisms. In *Saccharomyces cerevisiae*, furfural has been shown to inhibit glycolysis (Banerjee et al. 1981), and both furfural and HMF reduce bulk translational activity (Iwaki et al. 2013). In *Escherichia coli*, furfural depletes the cellular NADPH pool, leading to growth inhibition (Miller et al. 2009). It has also been reported to cause DNA damage (Hadi et al. 1989).

Adaptive laboratory evolution (ALE) has emerged as a powerful tool to increase the growth and fitness of microbes and can be used to attain a desirable microbial phenotype through directed natural selection under specific growth conditions. It has been successfully used to improve microbial tolerance to various stressors including thermal stress, oxidative stress, solvent stress, and low pH (Han et al. 2024; Papiran & Hamedi 2021; Su et al. 2025; Wang et al. 2020). ALE has also been successfully employed to enhance tolerance to lignocellulosic inhibitors in several industrial microorganisms. For example, ALE was used to improve furfural tolerance in *Escherichia coli*, where the tolerance was associated with metabolic rewiring and specific genomic mutations (Zheng et al. 2022), and to enhance lignocellulosic hydrolysate tolerance in *Pediococcus pentosaceus*, a lactic acid bacterium (Liu et al. 2021). These studies suggest various mechanisms of furfural and HMF adaptation, including suppression of energy-intensive processes such as efflux pumps to conserve energy, reduction of aldehydes to less toxic alcohol derivatives, metabolic rewiring to enhance ATP and NADPH supply, and global regulatory changes that improve stress tolerance. Additionally, attempts have been made to improve tolerance to lignocellulosic inhibitors by combining rational genetic engineering with ALE. For example, CREATE, a CRISPR-based approach was used to accelerate the adaptation of *E. coli* to furfural (Zheng et al. 2022). In a few cases, causal mutations in the furfural-adapted microbes have been identified by reverse engineering them in the wildtype strains. For example, ALE of *Pseudomonas putida* under furfural stress led to mutations in transporters, transcriptional regulators, and aldehyde dehydrogenases, and overexpression of these genes in the parental strain led to increased furfural tolerance (Zou et al. 2022).

Lactic acid, a versatile compound produced via fermentation by lactic acid bacteria (LAB), holds significant industrial importance, particularly for polylactic acid production (Rajendran et al. 2024). Among various LAB, *Lactiplantibacillus plantarum*, notable for its metabolic adaptability and stress tolerance, can efficiently utilize various sugars abundant in lignocellulose (Martino et al. 2016), making it a promising candidate for lactic acid production from lignocellulosic biomass (Alves de Oliveira et al. 2018).

In this study, we applied ALE to adapt *L. plantarum* JGR2, a strain previously isolated in our lab, to tolerate furfural and HMF- two major lignocellulosic growth inhibitors- with the aim of improving its performance in LCH. Furthermore, we investigated the underlying mechanisms of tolerance to these furans in the evolved mutants and evaluated the lactic acid production by the adapted strains in rice straw hydrolysate (RSH). To the best of our knowledge, this is the first study to adapt *L. plantarum* to these inhibitors with the specific objective of enhancing lactic acid production from lignocellulosic biomass.

## Materials and methods

### Cultivation of *L. plantarum*

The wild-type strain *L. plantarum* JGR2 (hereafter referred to as WT) was previously isolated in our laboratory (Surve et al. 2022). All strains were cultivated in modified de Man, Rogosa, and Sharpe (MRS) medium supplemented with 0.05% w/v L-cysteine (mMRS medium) and incubated at 37 °C under static conditions. For each experiment, precultures of all bacterial strains were initiated by transferring a single colony into mMRS broth, followed by incubation at 37 °C for 24 h. A 2% (v/v) inoculum from the preculture was subsequently transferred to fresh media under specific experimental conditions to establish the main culture.

### Adaptive laboratory evolution

ALE was initiated in three independent parallel populations for each condition. A preculture of WT was prepared, and 2% (v/v) inoculum was transferred to tubes containing 10 mL fresh mMRS broth, supplemented with either 3.5 g/L furfural or 2.5 g/L HMF. As described earlier (Surve et al. 2024a), these inhibitor concentrations caused about 33% growth inhibition of WT. Serial transfers of 2% (v/v) cultures into fresh inhibitor-supplemented media were performed every 24 h. The concentrations of the inhibitors were increased by 1.5-fold every three weeks. The transfers were carried out for 10 weeks, reaching final concentrations of 8 g/L furfural and 7 g/L HMF. Glycerol (15% v/v) stocks of the evolving populations were frozen every week. A passage control (PC) in which the WT was passaged for 10 weeks without any inhibitor (Surve et al. 2024b) was included for comparison in all the further assays. At the end of 10 weeks, two colonies were isolated from each terminal population. Tolerance of WT, evolving populations, two isolates from each terminal population (pure cultures), and one passage control, to the inhibitors (8 g/L furfural or 7 g/L HMF) was measured by monitoring the optical density at 600 nm (OD_600_).

### Whole genome resequencing and mutation analysis

Whole genome resequencing (WGS) and mutation analysis was carried out as described by us previously (Surve et al. 2024b).

### Transcriptomic analysis

One furfural-adapted isolate (F1a) and one HMF-adapted isolate (H2f) was selected for transcriptomic analysis. PC and adapted isolates were grown overnight in mMRS medium with or without their respective inhibitors (3.5 g/L furfural or 2.5 g/L HMF). Cultures were subsequently processed for transcriptome analysis, with all samples analyzed in biological duplicates, on the HiSeq 2500 platform (Illumina) with 2×150 chemistry which was outsourced. The quality of raw sequencing reads was assessed using FastQC (v0.12.1). Reads were mapped to the *L. plantarum* WCFS1 reference genome using Bowtie2 (v2.5.4), followed by sorting and indexing with SAMtools (v1.22.1). Transcript abundance was quantified using featureCounts (v2.1.1) with a GTF-format gene annotation file. Differential gene expression analysis for the PC and adapted strains was performed using the DESeq2 pipeline (Bioconductor). To account for multiple-testing, *p*-values were corrected using the Benjamini–Hochberg false discovery rate (FDR) correction. A cutoff of an adjusted *p*-value ≤ 0.05 and log2fold change ≥1 and ≤ − 1 was applied to define the final significant differentially expressed genes. Gene Ontology (GO) annotation of differentially expressed genes was performed using the Database for Annotation, Visualisation, and Integrated Discovery (DAVID) (https://davidbioinformatics.nih.gov) with a cutoff of adjusted *p-*value <0.05 (Huang et al. 2009; Sherman et al. 2022).

### Furfural biotransformation

Bacteria were grown overnight in mMRS broth (15 mL) supplemented with the respective inhibitors (3.5 g/L furfural or 2.5 g/L HMF). The cultures were centrifuged at 4500 ×g for 5 min at room temperature (RT), the cell-free supernatants (CFS) were extracted with equal volume of ethyl acetate and analyzed via Gas Chromatography-Mass Spectrometry (GC–MS) as described previously (Surve et al. 2024a). The results were expressed as the ratio of peak area of furfuryl alcohol to peak area of furfural quantified through Gas Chromatography-Flame Ionization Detector (GC-FID).

### Membrane fatty acid analysis

Bacteria were grown overnight in media with and without respective inhibitors (3.5 g/L furfural or 2.5 g/L HMF respectively). Fatty acid methyl ester (FAME) derivatization and analysis by GC–MS/FID was performed as described earlier (Surve et al. 2024b).

### Field emission scanning electron microscopy (FESEM)

WT, PC, and the adapted isolates were grown in respective stress conditions for 12 h (8 g/L furfural or 7 g/L HMF), and harvested by centrifugation, and washed with phosphate-buffered saline (PBS). The cell pellet was fixed in 2.5% glutaraldehyde and incubated at 4 °C for 2 h, followed by PBS washes. The fixed culture suspension was then coated onto poly L-lysine coated silicon wafers (P-type) and allowed to air-dry. Samples were dehydrated through a graded ethanol series (10% to 100%). The FESEM imaging was outsourced. Briefly, the dehydrated samples were sputter-coated with a thin gold layer, and visualized under a field emission scanning electron microscope at 15 kV, 30,000X to examine cell morphology.

### Assessment of lactic acid production in rice straw hydrolysate

RSH was sourced from Praj Industries Ltd, Pune, India. The composition of the hydrolysate as reported by the company was 3.6% glucose, 1.8% xylose, 0.13% acetic acid, 0.23% arabinose, and 0.02% HMF. Since the hydrolysate we obtained had little to no amount of HMF and furfural respectively, we prepared a RSH medium by dissolving 5 g/L yeast extract, 3 g/L furfural, and 2.5 g/L HMF in RSH and adding an equal volume of deionized water. The isolates were inoculated into the RSH medium at 10^6^ CFU/mL (colony forming units/ml) and incubated at 37 °C for 24 h under static conditions. Growth was measured by enumeration on mMRS agar. For estimating lactic acid levels, the culture supernatants were extracted with 5 mL of ethyl acetate containing 5 µg/mL of the internal standard (IS), tridecanoic acid (Ding et al. 2018). Trimethylsilyl ester (TMS) derivatization and GC–MS/FID analyses were performed as previously described (Surve et al. 2024b).

## Results and discussion

### Improved growth performance of adapted strains in the presence of the lignocellulosic inhibitors

**Fig. 1:**
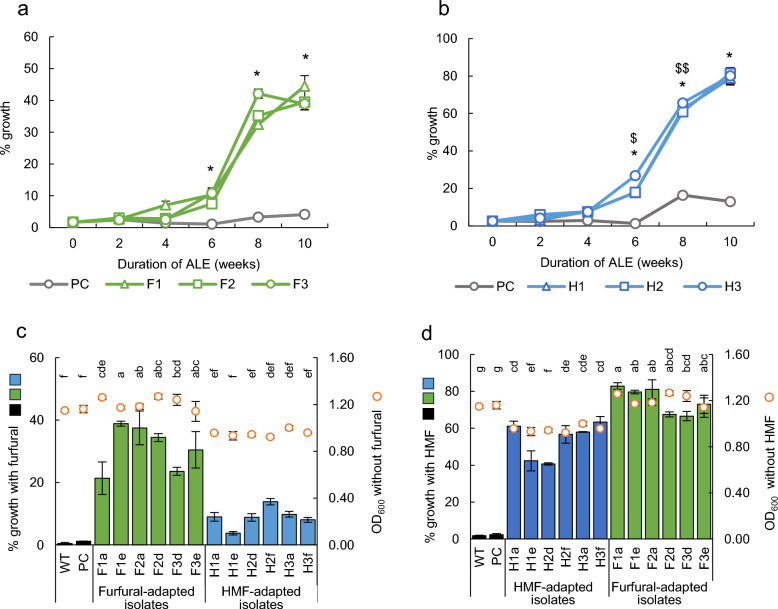
Development of tolerance to furfural and HMF in *L. plantarum* JGR2 through adaptive laboratory evolution (ALE). Growth in medium supplemented with 8 g/L furfural is shown for **a** weekly populations and **c** furfural-adapted isolates. Growth in medium supplemented with 7 g/L HMF is shown for **b** weekly populations and (d) HMF-adapted isolates. Growth data are presented for the wild type (WT), passage control (PC), weekly populations from furfural adaptation (F1, F2, F3), weekly populations from HMF adaptation (H1, H2, H3), furfural-adapted isolates (F1a, F1e, F2a, F2d, F3d, F3e), and HMF-adapted isolates (H1a, H1e, H2d, H2f, H3a, H3f). Growth is expressed as the percentage of the corresponding culture grown in inhibitor-free mMRS medium (OD₆₀₀), which was set to 100%. Error bars represent the standard error. Statistical analyses were performed to compare differences in growth among populations in the presence of inhibitors using one-way analysis of variance (ANOVA) followed by Tukey's test (p ≤ 0.05). For (**a**) and (**b**), * indicates a significant difference compared with WT and PC. $ indicates significantly higher growth of H3 than H1 and H2, while $$ indicates significantly higher growth of H3 than H2. No other significant differences among populations were observed in (**a**) and (**b**). For (**c**) and (**d**), different superscript letters above the bars indicate statistically significant differences between the samples while the samples that share at least one common letter are not significantly different from each other

ALE was commenced by growing *L. plantarum* JGR2 in the presence of 3.5 g/L furfural and 2.5 g/L HMF which causes 33% growth inhibition (Surve et al. 2024a). During the course of adaptation, the populations showed continuous increase in the tolerance to the respective inhibitors (Fig. 1). The week 10 cultures showed the highest growth (38% to 45% for furfural-adapted and 78% to 80% for HMF-adapted populations) in the presence of 8 g/L furfural and 7 g/L HMF, respectively. On the other hand, WT and PC showed maximum of 10% growth in the presence of these inhibitors (Fig. 1a, b). The OD_600_ values of the weekly populations is given in Table S1. Since the bacterial populations that have undergone ALE usually have a mixture of genetic variants, two pure cultures from each week 10 population were isolated. These pure cultures also exhibited a greater extent of tolerance to their respective inhibitors than that of the WT and PC (Fig. 1c, d). Specifically, furfural-adapted isolates exhibited 21.3–38.8% growth in the presence of furfural, while HMF-adapted isolates depicted 40.6–63.3% growth in the presence of HMF. This contrasts with the maximum 2.3% growth of WT or a PC isolate in the presence of furfural and HMF.

Earlier studies have used ALE to obtain bacterial strains tolerant to furfural and HMF. For instance, a related lactic acid bacterium *P. pentosaceus* adapted to furfural and HMF has been reported to tolerate up to 12.5 g/L and 11.5 g/L, of these chemicals, respectively (Liu et al. 2021). Consistent with this highly relevant benchmark, our adapted *L. plantarum* isolates successfully achieved comparable high-level tolerances of up to 8 g/L furfural and 7 g/L HMF.

Next, we were interested in understanding whether furfural- and HMF-adaptation leads to development of cross tolerance to HMF and furfural, respectively. Indeed, the isolates not just showed the development of such kind of cross-tolerance to the furans, but also, three furfural-adapted isolates showed greater tolerance to HMF than HMF-adapted isolates themselves (Fig. 1c, d). Both furfural and HMF have a reactive carbonyl group and thus similar mechanism of action of microbial growth inhibition. This includes generation of reactive oxygen species and subsequent cellular and biomolecular damage (Jilani & Olson 2023). The cross-tolerance that we observed is also consistent with earlier observation in case of *E. coli* (Wang et al. 2012) and *P. putida* (Zou et al. 2022). Furthermore, intra-chemical class cross-tolerance has also been reported in *E. coli* (Lennen et al. 2023).

### Mutations identified in furfural and HMF-adapted isolates

**Fig. 2:**
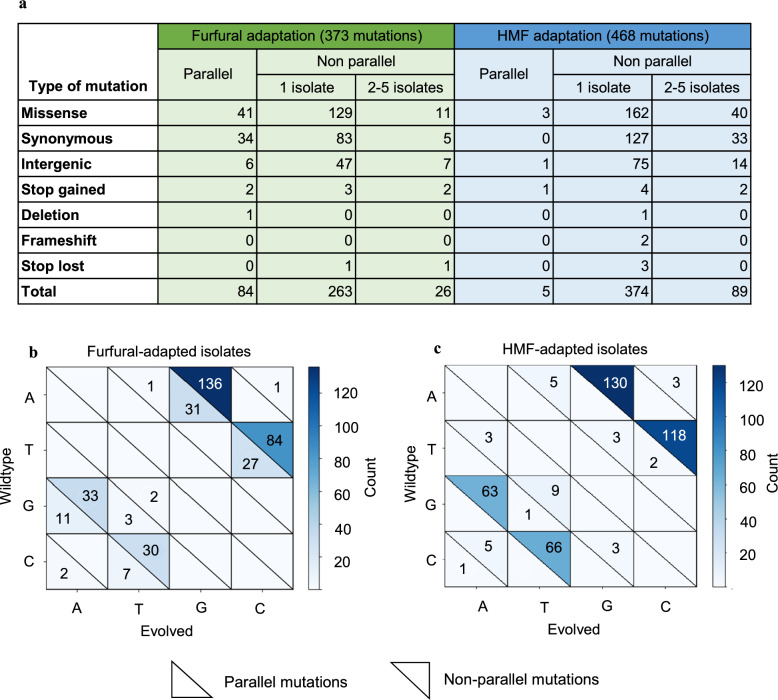
Cumulative distribution of mutation type in furfural- and HMF-adapted isolates of *L. plantarum* JGR2. **a** Number of different types of parallel and non-parallel mutations identified across the furfural- and HMF- adapted isolates, **b**, **c** Single nucleotide polymorphism (SNP) matrices showing the number of transitions and transversion mutations in (**b**) furfural-adapted isolates, and (**c**) HMF-adapted isolates. Complete numerical datasets for all identified mutations are provided in Tables S1 and S2

To identify the possible genomic mechanism of furfural and HMF adaptation, whole genome resequencing of the adapted isolates was performed, and the reads were mapped against the WT genome to identify the mutations. The mutations detected in PC (Surve et al. 2024b) were excluded. Collectively, a total of 373 and 468 mutations were observed in furfural and HMF-adapted isolates, respectively (Fig. 2a, Table S2, S3). In both the cases, missense mutations were the most dominant (about 49% to 44%) followed by synonymous mutations and single nucleotide variations in the non-coding regions (Fig. 2a). Both furfural and HMF adaptation showed the highest proportion of unique mutations (mutations found in only one of the six isolates, 70.5% and 79.9%, respectively). While furfural-adapted isolates had a large proportion of parallel mutations (mutations common to all six isolates, 22.5%), HMF adaptation revealed only a small proportion of parallel mutations (1.3%). Cumulatively, transition mutations outnumbered the transversion; and within transitions, A:T to G:C mutations dominated (Fig. 2b, c). A possible explanation for this is the action of furfural against the AT-rich regions rather than the GC-rich regions of DNA (Hadi et al., 1989).

We further used Grantham scores that classifies the missense mutations as conservative or non-conservative based on the nature of amino acid change (Grantham 1974). The Grantham score quantifies the dissimilarity between amino acids based on physicochemical properties such as side-chain composition and polarity. The higher the score, the greater the potential structural and functional impact of the substitution. Accordingly, we categorized nonsynonymous mutations as conservative (Grantham score ≤60) or non-conservative (Grantham score >60). Amongst the missense mutations in the furfural-adapted isolates, 20 were non-conservative mutations, indicating that the properties of proteins encoded by them might be affected to a greater extent (Table 1). The tyrosine to cysteine substitution exhibited the highest Grantham score (194) and it occurred in DNA recombination and repair protein (RecF, lp_0005), flavin prenyltransferase (UbiX, lp_0271), oligo-1,6-glucosidase (lp_0189), and a hypothetical protein (lp_2473). Tyrosine to cysteine change is particularly consequential, as the introduction of sulfur residues promotes disulfide bond formation, enhancing protein stability. Such structural modifications can be beneficial; for example, site-directed mutagenesis introducing cysteine residues has been shown to increase the insecticidal activity of a *Bacillus thuringiensis* secreted protein (Wang et al. 2022). Consistent with this, proteins with disulfide bonds are often subject to positive selection (Feyertag and Alvarez-Ponce 2017). Thus, the four genes carrying such mutation may play a role in the increased fitness of the adapted isolates to the inhibitor. RecF has been shown to be essential for adaptive mutation processes in *E. coli* (He et al. 2006). Although this protein has not been extensively studied in the context of ALE, it is well known for its role in stress response, for instance, contributing to UV resistance in *Mycobacterium smegmatis* (Gupta et al. 2013). Flavin prenyltransferase (UbiX, lp_0271) synthesizes prenylated flavin mononucleotide (prFMN), a critical cofactor required for the gallate decarboxylation activity of 3-octaprenyl-4-hydroxybenzoate carboxy-lyase (UbiD, lp_2945) (Jiménez et al. 2013). In *Cupriavidus basilensis*, UbiX and UbiD, which are linked to ubiquinone biosynthesis, have also been shown to participate in the degradation of HMF (Koopman et al. 2010). However, we found a mutation in this gene in response to furfural adaptation, suggesting a potential role of this protein in furfural tolerance as well.

**Table 1:** List of parallel mutations across six furfural- and six HMF-adapted isolates of *Lactiplantibacillus plantarum* JGR2

Position	*L. plantarum* JGR2 gene ID	*L. plantarum* WCFS1 ortholog		Strand direction	Mutation	Amino acid change	Grantham score	Function	Type of mutation
		Locus tag	Gene name						
Furfural adaptation									
13,895	fig|1590.2119.peg.14	lp_0005	*recF*	→	A→ G	Y44C	194	DNA recombination and repair protein RecF	Missense
38,565	fig|1590.2119.peg.33	lp_0024	*glgP*	→	A→ G	N505S	46	Glycogen phosphorylase	Missense
47,299	fig|1590.2119.peg.41	lp_0032		→	A→ G	K10R	26	hypothetical protein	Missense
107,031	fig|1590.2119.peg.103	lp_0104	*larA*	→	A→G	L332L	–	LarA: implicated in lactate racemization	Synonymous
115,632	fig|1590.2119.peg.113	lp_0116	*thiP*	→	G→ A	G299E	98	Predicted hydroxymethylpyrimidine transporter CytX	Missense
156,780	fig|1590.2119.peg.156	–		→	C→T	P235P	–	Maltodextrin glucosidase	Synonymous
184,309	fig|1590.2119.peg.175	lp_0189		→	A→ G	Y500C	194	Oligo–1,6–glucosidase	Missense
191,682	fig|1590.2119.peg.181	lp_0197		→	Δ12 bp	S78_S82del	–	hypothetical protein	Inframe–deletion
254,261	fig|1590.2119.peg.244	lp_0271		→	A→ G	Y78C	194	Flavin prenyltransferase UbiX	Missense
256,647	fig|1590.2119.peg.247	lp_0274		→	G→ A	A228T	58	Transcriptional regulator, AcrR family	Missense
287,422	fig|1590.2119.peg.278	lp_0305	*gcsH1*	←	T→ C	H23R	29	hypothetical protein	Missense
357,014	fig|1590.2119.peg.350	–		→	A→G	N86D	23	hypothetical protein	Missense
381,971	fig|1590.2119.peg.383	lp_0423	*plnG*	→	A→G	T31T	–	Competence–stimulating peptide ABC transporter ATP–binding protein ComA	Synonymous
441,291	fig|1590.2119.peg.440	lp_0502	*sdaC*	→	A→G	S183S	–	Serine transporter	Synonymous
533,900	fig|1590.2119.peg.518	lp_0589	*accB1*	→	A→G	V42V	–	hypothetical protein	Synonymous
573,972	fig|1590.2119.peg.559	lp_0700	*recR*	→	G→ A	G37S	56	Recombination protein RecR	Missense
693,222	–	–	*–*	–	T→C	–	–	–	Intergenic
765,367	fig|1590.2119.peg.756	lp_1106	*citC*	→	A→ G	I46V	29	[Citrate [pro–3S]–lyase] ligase	Missense
831,071	fig|1590.2119.peg.814	lp_1171	*pbpX1*	→	A→G	K41K	–	hypothetical protein	Synonymous
866,723	fig|1590.2119.peg.845	lp_0781		→	A→ G	I181V	29	Sporulation transcription regulator WhiA	Missense
869,904	fig|1590.2119.peg.847	lp_0785		←	G→ A	Q159*	–	Glyoxylate reductase (EC 1.1.1.79) @ Glyoxylate reductase (EC 1.1.1.26) @ Hydroxypyruvate reductase (EC 1.1.1.81)	Stop–gained
887,191	fig|1590.2119.peg.861	lp_0800		→	G→ A	A265T	58	hypothetical protein	Missense
924,803	fig|1590.2119.peg.895	lp_0835		→	G→ A	R5H	29	hypothetical protein	Missense
1,003,034	fig|1590.2119.peg.967	lp_0923		→	A→ G	I337V	29	hypothetical protein	Missense
1,037,042	fig|1590.2119.peg.1002	lp_1251		←	C→ T	D156N	23	6–phosphogluconate dehydrogenase, decarboxylating	Missense
1,103,421	fig|1590.2119.peg.1051	lp_1312	*tagE3*	→	A→G	Q10Q	–	Poly(glycerol–phosphate) alpha–glucosyltransferase	Synonymous
1,198,447	fig|1590.2119.peg.1140	lp_1435		←	G→ A	T677I	89	Uncharacterized membrane protein YfhO–like	Missense
1,220,642	fig|1590.2119.peg.1162	lp_1458	*trmB*	→	A→ G	K6R	26	tRNA (guanine(46)–N(7))–methyltransferase	Missense
1,372,375	fig|1590.2119.peg.1312	lp_1660		→	G→ A	R267H	29	Quinone oxidoreductase	Missense
1,382,356	fig|1590.2119.peg.1323	lp_1673	*fabD*	→	A→G	V45V	–	Malonyl CoA–acyl carrier protein transacylase	Synonymous
1,409,311	fig|1590.2119.peg.1359	lp_1711	*pepD4*	←	T→C	L393L	–	Dipeptidase	Synonymous
1,448,554	fig|1590.2119.peg.1394	lp_1751	*pbp1A*	←	A→ G	Y242H	83	Multimodular transpeptidase–transglycosylase / Penicillin–binding protein 1A/1B (PBP1)	Missense
1,462,709	fig|1590.2119.peg.1410	–		←	T→C	G64G	–	hypothetical protein	Synonymous
1,464,908	fig|1590.2119.peg.1412	lp_1776		→	A→ G	D267G	94	Glycine/D–amino acid oxidases family	Missense
1,527,630	fig|1590.2119.peg.1478	lp_1845	*hslU*	←	T→ C	E433G	98	ATP–dependent hsl protease ATP–binding subunit HslU	Missense
1,546,597	fig|1590.2119.peg.1494	lp_1865		←	T→C	Q32Q	–	UPF0346 protein YozE	Synonymous
1,657,093	fig|1590.2119.peg.1604	lp_1983		→	T→C	H179H	–	Ribulosamine/erythrulosamine 3–kinase potentially involved in protein deglycation	Synonymous
1,692,733	fig|1590.2119.peg.1638	lp_2040	*infB*	←	G→A	D355D	–	Translation initiation factor 2	Synonymous
1,745,490	fig|1590.2119.peg.1693	lp_2100	*cps4I*	←	T→C	Q226Q	–	hypothetical protein	Synonymous
1,746,955	fig|1590.2119.peg.1694	lp_2101	*cps4H*	←	T→ C	I158V	29	hypothetical protein	Missense
1,750,678	fig|1590.2119.peg.1698	lp_2105	*cps4D*	←	T→C	A158A	–	UDP–glucose 4–epimerase	Synonymous
1,794,589	fig|1590.2119.peg.1738	lp_2151	*pdhD*	←	A→G	D125D	–	Dihydrolipoamide dehydrogenase of pyruvate dehydrogenase complex	Synonymous
1,844,377	fig|1590.2119.peg.1785	lp_2211	*cspR*	←	A→G	H61H	–	tRNA (cytidine(34)–2'–O)–methyltransferase	Synonymous
1,899,045	fig|1590.2119.peg.1847	lp_2277	*alaS*	←	A→ G	L515S	145	Alanyl–tRNA synthetase	Missense
1,920,672	fig|1590.2119.peg.1863	lp_2300		←	C→ T	D480N	23	Ribonuclease Y	Missense
1,944,509	fig|1590.2119.peg.1884	lp_2323	*tpx*	←	T→C	V39V	–	Thiol peroxidase, Tpx–type	Synonymous
1,945,368	fig|1590.2119.peg.1886	lp_2324	*gshA*	←	A→G	N214N	–	Glutathione biosynthesis bifunctional protein gshF	Synonymous
1,945,412	fig|1590.2119.peg.1886	lp_2324	*gshA*	←	C→ A	A200S	99	Glutathione biosynthesis bifunctional protein gshF	Missense
1,945,829	fig|1590.2119.peg.1886	lp_2324	*gshA*	←	A→G	L61L	–	Glutathione biosynthesis bifunctional protein gshF	Synonymous
1,945,884	fig|1590.2119.peg.1886	lp_2324	*gshA*	←	C→T	S42S	–	Glutathione biosynthesis bifunctional protein gshF	Synonymous
2,008,833	fig|1590.2119.peg.1949	lp_2395		←	A→G	H336H	–	Efflux ABC transporter, permease/ATP–binding protein EF2593	Synonymous
2,021,924	fig|1590.2119.peg.1959	–		←	A→G	S770S	–	Phage tail length tape–measure protein T	Synonymous
2,023,852	fig|1590.2119.peg.1959	–		←	T→C	K128E	56	Phage tail length tape–measure protein T	Missense
2,057,312	fig|1590.2119.peg.2007	lp_2466		←	T→ C	I264M	10	hypothetical protein	Missense
2,061,134	fig|1590.2119.peg.2014	lp_2473		←	T→ C	Y385C	194	hypothetical protein	Missense
2,099,872	fig|1590.2119.peg.2059	lp_2525		←	T→ C	N466D	23	Bis–ABC ATPase SPy1206	Missense
2,101,895	fig|1590.2119.peg.2060	lp_2526		←	T→ C	I15M	10	hypothetical protein	Missense
2,101,976	–	–	*–*	–	T→C	–	–	–	Intergenic
2,206,233	fig|1590.2119.peg.2161	lp_2658		←	A→ G	V101A	64	hypothetical protein	Missense
2,213,584	fig|1590.2119.peg.2168	lp_2665	*rrp8*	←	T→ C	E192G	98	hypothetical protein	Missense
2,254,458	–	–	*–*	–	C→T	–	–	–	Intergenic
2,391,227	fig|1590.2119.peg.2339	lp_2862	*sip1*	→	T→C	H142H	–	Signal peptidase I	Synonymous
2,431,881	fig|1590.2119.peg.2379	lp_2915	*uvrA3*	→	T→C	S441S	–	Excinuclease ABC subunit A paralog of unknown function	Synonymous
2,443,280	–	–	*–*	–	G→A	–	–	–	Intergenic
2,444,614	fig|1590.2119.peg.2388	lp_2925		←	T→ C	Q350R	43	Cell surface protein	Missense
2,464,353	fig|1590.2119.peg.2409	lp_2948		→	G→T	S50S	–	hypothetical protein	Synonymous
2,609,452	fig|1590.2119.peg.2555	lp_3108		←	T→ C	H203R	29	hypothetical protein	Missense
2,619,668	fig|1590.2119.peg.2569	–		→	T→C	G33G	–	Transcriptional regulator, MerR family	Synonymous
2,623,328	fig|1590.2119.peg.2573	–		←	A→G	M1421T	81	hypothetical protein	Missense
2,631,594	fig|1590.2119.peg.2577	–		→	C→A	P60Q	76	Mobile element protein	Missense
2,631,603	fig|1590.2119.peg.2577	–		→	G→T	R63L	102	Mobile element protein	Missense
2,631,613	fig|1590.2119.peg.2577	–		→	CCCAA→TCCAC	SPR66SSPR	–	Mobile element protein	Synonymous
2,631,673	fig|1590.2119.peg.2577	–		→	A→G	K86K	–	Mobile element protein	Synonymous
2,631,694	fig|1590.2119.peg.2577	–		→	C→T	S93S	–	Mobile element protein	Synonymous
2,631,704	fig|1590.2119.peg.2577	–		→	G→A	V97I	29	Mobile element protein	Missense
2,631,832	fig|1590.2119.peg.2577	–		→	T→C	D139D	–	Mobile element protein	Synonymous
2,631,842	fig|1590.2119.peg.2577	–		→	AGACAA→CGACGG	RQ143RR	43	Mobile element protein	Missense
2,631,862	fig|1590.2119.peg.2577	–		→	A→G	S149S	–	Mobile element protein	Synonymous
2,631,904	fig|1590.2119.peg.2577	–		→	G→T	L163L	–	Mobile element protein	Synonymous
2,713,664	fig|1590.2119.peg.2666	lp_3254	*lrgA*	←	T→ C	N131D	23	hypothetical protein	Missense
2,835,852	–	–	*–*	–	A→G	–	–	–	Intergenic
2,877,027	fig|1590.2119.peg.2830	lp_3476	*ramR*	→	C→ T	Arg320*	–	Transcriptional regulator of rhamnose utilization, AraC family	Stop–gained
2,890,048	–	–	*–*	–	T→C	–	–	–	Intergenic
3,029,029	fig|1590.2119.peg.2980	lp_3661	*rbsR*	←	T→C	P193P	–	Ribose operon repressor	Synonymous
HMF adaptation									
388,011	fig|1590.2119.peg.389	lp_0429	*plnW*	→	C→A	Q68K	53	hypothetical protein	Missense
1,374,675	fig|1590.2119.peg.1314	lp_1663		→	G→T	A405S	99	hypothetical protein	Missense
2,644,587	fig|1590.2119.peg.2591	lp_3173		→	T→C	S245G	56	hypothetical protein	Missense
2,989,013	fig|1590.2119.peg.2935	–		←	G→A	Trp122*	–	hypothetical protein	Stop–gained

Some of the cell membrane/ cell surface proteins were also found to have non-synonymous mutations in furfural and HMF-adapted isolates. For furfural-adapted isolates, these include *lp_1435* (encoding an uncharacterized membrane protein), *lp_1751* (*pbp1A*, encoding multimodular transpeptidase-transglycosylase / penicillin-binding protein 1A/1B) and *lp_2925* (encoding a cell surface protein), along with an intergenic mutation upstream of *lp_1010* (*dacB*, D-alanyl-D-alanine carboxypeptidase, a cell surface protein) (Table 1). Three of the only five parallel mutations observed in HMF-adapted isolates were also in the putative cell membrane or cell surface proteins (lp_0429, lp_1663, and lp_3173). These observations suggest that cell surface modulations might represent a potential mechanism of furan tolerance observed in these isolates. Such mutations in cell wall-associated proteins during adaptation to industrial chemicals has also been reported in *E. coli* (Lennen et al. 2023).

### Transcriptomic responses to furfural and HMF stress and adaptation

**Fig. 3:**
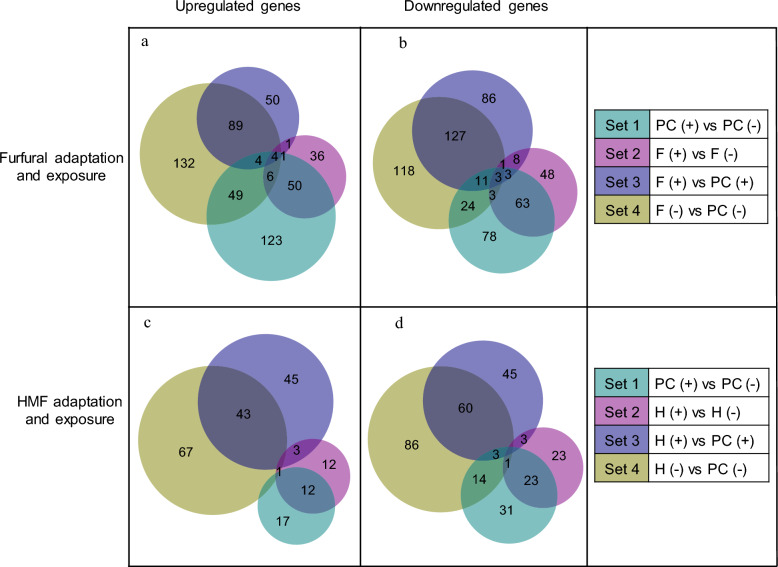
Venn diagrams showing the number of differentially expressed genes (DEGs) in *L. plantarum* JGR2 after adaptation and exposure to furfural and HMF. For transcriptomic analysis, the passage control was cultured in the absence [PC (-)] and presence [PC ( +)] of inhibitors, while adapted isolates were grown either without [F (-) or H (-)] or with their respective inhibitor [F ( +) or H ( +)]. (a,b) DEGs associated with furfural adaptation and exposure. (c,d) DEGs associated with HMF adaptation and exposure. Panels (**a**, ** c**) show upregulated genes, while panels (**b**, **d**) show downregulated genes. Numbers in overlapping regions represent genes shared between the respective comparison sets. A cutoff of adjusted p-value ≤ 0.05 and log2fold change ≥1 and ≤ − 1 was applied

To get insights into the transcriptomic mechanisms of furfural and HMF tolerance, the adapted isolates were each subjected to RNA-seq analyses upon growing them under various conditions. Considering consistent inhibitor tolerance depicted by all the adapted isolates, and the presence of several parallel mutations, as detailed above, only one representative furfural- and HMF- adapted isolate were used for this purpose. Specifically, PC was grown in the absence [PC (–)] and presence [PC (+)] of inhibitors, while adapted isolates were cultured either without [F (–) or H (–)] or with their respective inhibitor [F (+) or H (+)] for transcriptomic profiling. Principal component analysis (PCA) plot revealed distinct clustering of biological replicates, indicating high reproducibility (Fig. S1). Clear separation of furfural-adapted isolates along PC1 suggests the pronounced effect of furfural adaptation on global gene expression patterns. Separation along PC2 reflected treatment-specific transcriptional responses to exposure to furfural and HMF. Global transcriptional differences across conditions are visualized using hierarchical clustering of normalized transcripts per million values (Fig. S2). The heatmap reveals clear separation between adapted and parental samples, indicating a distinct transcriptional profile following adaptation. Differential gene expression analysis was conducted using four comparison sets for each adaptation: PC (+) vs PC (–), F/H (+) vs F/H (–), F/H (+) vs PC (+), and F/H (–) vs PC (–), hereafter referred to as sets 1, 2, 3, and 4 respectively (Fig. S3). Sets 1 and 2 reflect the transcriptional effects of inhibitor treatment, whereas sets 3 and 4 capture changes associated with adaptive evolution. Venn diagrams illustrate the number of differentially expressed (DE) genes that are unique to, or shared between, these sets (Fig. 3). A list of all differentially expressed genes is provided in Table S4. In the furfural-adapted isolate, sets 1, 2, 3, and 4 comprised 422, 227, 388, and 571 DE genes, respectively (Fig. 3a, b). Similarly, in the HMF-adapted isolate, the respective sets contained 102, 78, 203, and 275 DE genes (Fig. 3c, d). Overall, higher number of DE genes were found in each furfural sets than the corresponding HMF sets. In case of both furfural and HMF, the numbers of DE genes upon adaptation (sets 3 and 4) were higher than those upon the inhibitor exposure (sets 1 and 2), indicating that adaptation is associated with more extensive changes in the transcriptional profile than acute inhibitor exposure. Upon exposure to furfural, the furfural-adapted isolate exhibited fewer DE genes (set 2) compared to PC (set 1), suggesting a reduced requirement for transcriptional adjustment in the adapted strains. However, this pattern was not observed in the HMF-adapted isolate indicating a differing transcriptional response compared to furfural adaptation.

Gene Ontology (GO) enrichment analysis was performed to identify biological processes, molecular functions, and cellular components overrepresented among the differentially expressed genes (Table S5). Upon furfural exposure, the passaged control (Set 1 of furfural adaptation) exhibited significant enrichment of GO categories associated with amino acid, organic acid, and nucleotide biosynthesis and metabolism, in addition to translation- and ribosome- related processes. In comparison, HMF exposure (Set 1 of HMF adaptation) enriched fewer GO categories, which were primarily associated with amino acid and organic acid biosynthesis and metabolism. Translation- and ribosome-related GO terms were enriched only in the passaged control following furfural exposure and not in the adapted isolate (Set 2 of furfural adaptation). No significantly enriched GO terms were observed in Sets 3, and 4 of furfural adaptation and sets 2, 3, and 4 of HMF adaptation. Overall, GO enrichment analysis indicated a broader functional response to furfural than to HMF in the passaged control, whereas the furfural-adapted isolate exhibited enrichment of fewer functional categories, largely restricted to core metabolic and biosynthetic processes. These observations are consistent with the RNA-seq results (Fig. 3) showing fewer differentially expressed genes in the adapted strains following inhibitor challenge.

Furfural and HMF adaptation resulted in a 1.17 and 1.18-fold upregulation, respectively, of *lp_3051* (*dhaT* 1,3-propanediol dehydrogenase) compared to PC. We have previously shown that *L. plantarum dhaT* encodes for furfural reductase activity that converts furfural into less noxious furfuryl alcohol, and that *dhaT* is upregulated upon furfural exposure in WT (Surve et al. 2024a). Although furfural exposure also induced *dhaT* expression in the furfural-adapted isolates, the magnitude of induction (3.84-fold) was lower than that observed in PC upon furfural exposure (4.48-fold). Together, these observations suggest that furfural adaptation may precondition the cells for detoxification by maintaining constitutively elevated *dhaT* expression.

A locus *lp_0746*-*lp_0751* (*pstABCD* operon) involved in phosphate uptake was constitutively upregulated in the furfural-adapted isolates in addition to its upregulation in PC upon furfural exposure (*lp_0747-lp_0751*). In *Lactococcus lactis*, the *pst* operon acts as a major hub for cross-resistance against toxic carbonyl compounds and metabolic stressors, such as acetoin and methylglyoxal (Cesselin et al. 2021). This suggests that phosphate uptake and metabolism could serve as a possible mechanism of response to noxious chemicals in *L. plantarum*, requiring future targeted studies. Additionally, a few other uncharacterized ABC transporters and MFS (major facilitator superfamily) transporters were constitutively upregulated in furfural adapted isolates (Table S4), pointing towards their possible role in furfural tolerance, although direct evidence remains to be established. Upregulation of such transporters has also been previously observed during furfural adaptation in *P. putida* (Zou et al. 2022). In the HMF-adapted isolates, however, the number of downregulated transporters was higher than those which were upregulated (Table S4).

*Lp_0367* (*choS,* glycine betaine/carnitine/choline ABC transporter, substrate binding and permease protein) and *lp_0368* (*choQ*, glycine betaine/carnitine/choline ABC transporter, ATP-binding protein) were constitutively downregulated in the furfural-adapted isolate. Since such transporters are required for osmoprotection, their observed downregulation in furfural-adapted isolates could be a potential strategy to conserve ATP by inhibiting non-essential transport processes, a hypothesis that aligns with observations in furfural-adapted *E. coli* (Zheng et al. 2022).

While glycolytic genes such as *lp_0789* (*gapB*, glyceraldehyde 3-phosphate dehydrogenase), and *lp_0790* (*pgk*, phosphoglycerate kinase) were downregulated by furfural exposure in PC and the furfural-adapted isolate, furfural-adaptation resulted in their upregulation suggesting a possible compensatory mechanism. Upregulation of *lp_0849* (*pox1*, pyruvate oxidase) and *lp_2242* (*ack3*, acetate kinase) observed upon furfural-adaptation might be a strategy to obtain additional ATP. Downregulation of *lp_2629* (*pox3,* pyruvate oxidase) in the furfural-adapted isolate and upon HMF exposure in the HMF-adapted isolate, possibly suggests isoform-specific regulation of pyruvate kinase which has not yet been studied in *L. plantarum*. Downregulation of *lp_3551*(*xfp*, xylulose−5-phosphate phosphoketolase), *lp_3538* (*tkt4*, transketolase), and *lp_3539* (*tal2*, transaldolase) along with a D156N mutation in *lp_1251* (*gntZ*, 6‑phosphogluconate dehydrogenase) possibly indicates downregulation of pentose metabolism during furfural adaptation. Some of these observations are consistent with the redirection of central carbon metabolism observed in *E. coli* when furfural tolerance was imparted by CRISPR-enabled trackable genome engineering (Zheng et al. 2022). Several PTS (phosphotransferase system)/sugar uptake regulons appear to be strongly downregulated in furfural-adapted isolates. These mainly include ccpA-controlled PTS/sugar catabolic genes *lp_3507*-*lp_3513* (cellobiose, β-glucosides, sorbitol, galactitol, arabitol, N acetylglucosamine cluster), and MalR3 regulon (*lp_3530*-*lp_3534*). The inverse correlation between PTS activity and stress tolerance is known in bacteria (Zhu et al. 2024), and could possibly explain the above observations.

Several stress response genes such as *lp_0728* (*groEL*, GroEL chaperonin), *lp_0997* (*cspC*, cold shock protein), *lp_0031* (*cspL*, cold shock protein), *lp_0129* (*hsp1*, small heat shock protein), *lp_2668* (*hsp2*, small heat shock protein), and *lp_3352 (hsp3*, small heat shock protein) were constitutively upregulated in the furfural-adapted isolates. Two clp system genes, *lp_0786* (*clpP*, endopeptidase) proteolytic subunit and *lp_1269* (*clpE*, ATP-dependent Clp protease, ATP-binding subunit) were upregulated while another chaperon, *lp_3583* (*clpL,* ATP-dependent Clp protease) was strongly downregulated which may indicate the activation of a specific stress response. On the other hand, other than a downregulation in *lp_0129* (*hsp1*, small heat shock protein), *lp_0786* (*clpP*, endopeptidase), these genes were not differentially expressed in HMF adaptation. Genes encoding similar proteins such as GroESL, have been previously linked to furfural tolerance in *E. coli* (Jilani & Olson 2023).

A few two-component signaling (TCS) genes were downregulated in the furfural-adapted isolate. These include three genes of the *lamBDCA* operon, *lp_3582, lp_3581, lp_3580* which were strongly downregulated (minimum 7.62 folds), and *lp_3088-lp_3087* (*lamKR* operon) which was modestly downregulated (maximum 2.28 folds). Two-component systems aid in stress tolerance, like bile stress tolerance in *L. plantarum* (Chen et al. 2024). Several genes which have previously been reported to be downregulated in Δ*lamA* Δ*lamR* mutant (Fujii et al. 2008) were also downregulated in our study. Considering that these genes were involved in stress response, cell membrane integrity, and the metabolism of carbohydrates and lipids, *lamKR* operon might be involved in furfural adaptation possibly via regulating these functions.

Many of the above-mentioned genes downregulated in the furfural-adapted isolate were located in the region *lp_3489-lp_3583*. Indeed, nearly half of the genes in these regions were extensively downregulated. At the same time, this isolate along with two other furfural-adapted isolates also had a K719E mutation in RNA polymerase beta subunit (lp_1021, RpoB). RpoB mutations are known to be involved in bacterial adaptation in various stresses including furfural adaptation in *E. coli* (Zheng et al. 2022). Rodŕiguez-Verdugo et al (2016) found that adaptive mutation in *rpoB* correlated to the extensive transcriptomic changes in *E. coli*. Thus, the downregulation of several genes in the *lp_3489-lp_3583* region that we observed might also be a pleotropic effect potentially associated with the K719E mutation in RpoB.

The furfural-adapted isolate subjected to RNA-seq analysis (F1a) carried 84 and 58 missense and synonymous mutations (Table S2). Of all the genes carrying these mutations, 27 and 14 were differentially expressed in the same isolate compared to the PC (set 3 or 4), respectively (Table S4). Similarly, the HMF-adapted isolate subjected to RNA-seq analysis (H2f) carried 74 and 84 missense and synonymous mutations (Table S3). Two genes carrying each type of mutation were differentially expressed in this isolate compared to PC. Although synonymous mutations do not alter the functions of the encoded proteins, they are known to impact the expression of genes carrying these mutations (Bailey et al. 2021).

Some such genes which carried synonymous mutations and which were differentially expressed in the furfural-adapted isolate include a set of genes involved in capsular polysaccharide (CPS) biosynthesis, viz., *lp_2100* (*cps4I* glycosyltransferase, family 2), *lp_2102* (*cps4G*, glycosyltransferase, family 1), and *lp_2105* (*cps4D,* UDP N-acetyl glucosamine 4-epimerase, NAD dependent). These genes are a part of *cps4A-J* gene cluster (Remus et al. 2012). Interestingly, a few genes of *cps1A-I* cluster (*glf1*, *cps1A*, *cps1B*, *cps1D*) were downregulated in furfural-adapted isolates. Given that *cps1A-I* is involved in dictating the molecular weight and monosaccharide composition of the polysaccharides, and *cps4A-J* in determining the polysaccharide yield (Remus et al. 2012), constitutive differential regulation of these genes upon furfural adaptation suggests a possible role for specific surface polysaccharides in furfural tolerance, though this warrants further functional verification.

Similarly, *gshA* (*lp_2324*) encoding glutamate-cysteine ligase, a key enzyme in the glutathione biosynthesis pathway had multiple synonymous mutations (Table 1) and was also significantly downregulated in the furfural-adapted isolate (Table S4). Glutathione has been reported to play an important role in furfural tolerance in *Saccharomyces cerevisiae* (Jilani & Olson 2023). In *Listeria monocytogenes*, *gshF*, which encodes an enzyme involved in glutathione biosynthesis, is strongly upregulated under oxidative stress, and deletion of this gene results in impaired bacterial growth (Chen et al. 2023). Although our observation is in contrast with this study, it suggests that synonymous mutations in *gshA* may contribute to adaptation, potentially through effects on gene regulation.

### Adapted isolates reduce furfural to furfuryl alcohol

**Fig. 4:**
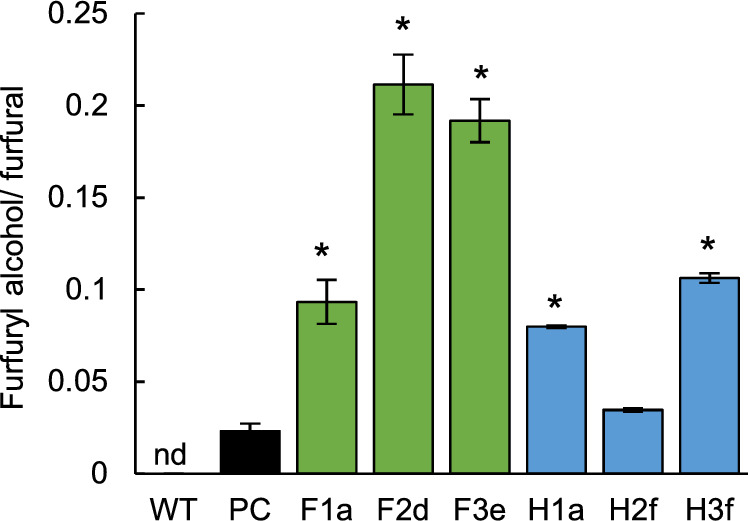
Furfuryl alcohol production by furfural and HMF-adapted *L. plantarum* JGR2. The ratio of furfuryl alcohol to furfural was determined after incubating wild-type (WT), passage control (PC), furfural-adapted isolates (F1a, F2d, and F3e) and HMF-adapted isolates (H1a, H2f, and H3f) with furfural, as determined by gas chromatography. WT showed no detectable production of furfuryl alcohol. Statistical analyses were performed by Kruskal–Wallis test followed by post-hoc Dunn’s test (*p* ≤ 0.05); *indicates significant difference from PC

Given the constitutive upregulation of *dhaT* encoding furfural reductase activity (Surve et al. 2024a), in furfural and HMF-adapted isolates, we sought to analyze the possible higher extent of biotransformation of furfural by the adapted isolates than the PC and WT. While WT did not show production of furfuryl alcohol under the given experimental conditions, furfural-adapted isolates clearly showed furfuryl alcohol production with 4 to 9.1 times higher furfuryl alcohol/ furfural ratio than that observed for PC (Fig. 4). HMF-adapted isolates also produced furfuryl alcohol, potentially explaining their cross-tolerance to furfural observed in prior experiments.

Constitutive upregulation of *dhaT* in both furfural and HMF-adapted isolates could possibly be a reason for the higher extent of furfural alcohol production by these isolates. In line with our observations, conversion of aldehydes into their respective alcohols has been reported earlier as an adaptive microbial response. For instance, syringaldehyde adaptation in *Lipomyces starkeyi* led to enhanced conversion of syringaldehyde to syringyl alcohol (Putra et al. 2024). Similarly, higher extent of furfural to furfuryl alcohol conversion was observed in *P*. *pentosaceus* upon domestication to LCH containing furan inhibitors (Liu et al. 2021). Thus, the observed enhanced biotransformation ability serves as a direct phenotypic validation of the constitutive *dhaT* upregulation observed in our transcriptomic data. Future analysis of furfuryl alcohol production kinetics in our furfural-adapted *L. plantarum* could provide details such as the rate and efficiency of furfural detoxification, which are key to understanding the mechanistic details.

### Membrane fatty acid modulation

**Fig. 5:**
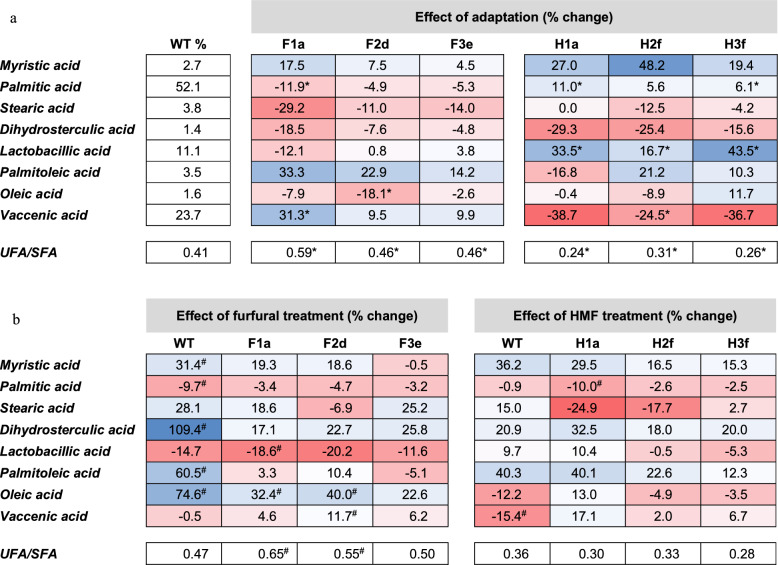
Membrane fatty acid modulation in *L. plantarum* JGR2 following adaptation and exposure to furfural and HMF. **a** Percentage fatty acid composition of the wild-type strain (WT) and the effect of adaptation on fatty acid profiles in furfural-adapted (F1a, F2d, and F3e) and HMF-adapted (H1a, H2f, and H3f) isolates. All values were obtained from cultures grown in inhibitor-free medium. The WT column shows the actual percentage composition of each fatty acid, while values for adapted isolates are expressed as percentage change relative to the corresponding fatty acid content of WT grown in inhibitor-free medium. Blue shading denotes increase in the proportion of that fatty acid compared to WT whereas red shading denotes a decrease. **b** Effect of inhibitor treatment on fatty acid composition in WT and adapted isolates. For furfural treatment, WT and furfural-adapted isolates were grown in the presence of furfural, whereas for HMF treatment, WT and HMF-adapted isolates were grown in the presence of HMF. Values are expressed as percentage change relative to the fatty acid content of the corresponding isolate grown in inhibitor-free medium. Blue shading denotes increase in the proportion of that fatty acid by the inhibitor compared to the inhibitor-free medium whereas red shading denotes a decrease. UFA/SFA denotes the ratio of unsaturated to saturated fatty acids. Statistical analyses were performed by one-way ANOVA followed by Tukey’s test (*p* ≤ 0.05); *indicates significant difference between WT and adapted isolates grown without inhibitor, and # indicates significant difference between the same isolate grown with and without inhibitor stress

Membrane fatty acid modulation is a well-documented bacterial stress tolerance mechanism (Willdigg and Helmann 2021). Hence, we studied the effects of furfural and HMF adaptation on the membrane fatty acid composition. A total of eight fatty acids were identified across all strains and were classified into unsaturated fatty acids (UFA) (palmitoleic, oleic, and vaccenic acids) and saturated fatty acids (SFA) (myristic, palmitic, stearic, dihydrosterculic, and lactobacillic acids) (Fig. 5). All furfural-adapted isolates showed a significant increase in the UFA/SFA ratio. These changes were attributed to a variable decrease in both palmitic and oleic acids, accompanied by an increase in vaccenic acid. Similar increases in the UFA/SFA ratio have been reported in *L. plantarum* LM1001 exposed to acid stress (Lee et al. 2024) and in *Lactobacillus acidophilus* strains that were adapted to acid stress (Nyabako et al. 2020). An increased UFA/SFA ratio enhances membrane fluidity, a feature that has been associated with improved tolerance to various stresses in bacteria (Fonseca et al. 2019). The elevated UFA/SFA ratio observed in the furfural-adapted isolates therefore indicates a significant shift in membrane composition, promoting greater fluidity and stress resistance. This adjustment likely represents one of the key mechanisms underlying adaptation to furfural.

On the other hand, all three HMF-adapted isolates uniquely over-accumulated lactobacillic acid with a concomitant decrease in the vaccenic acid in two isolates. This inverse correlation is obvious considering that lactobacillic acid is a cyclopropane fatty acid (CFA) formed from vaccenic acid. Increases in lactobacillic acid levels are a well-documented response to adverse environmental conditions in lactobacilli, and have been reported in acid adapted *Lactobacillus casei* (Broadbent et al. 2010). CFAs are associated with enhanced stress tolerance in *L. lactis* (Velly et al. 2015) and *E. coli* (Chen et al. 2016). These changes along with an increase in palmitic acid in some isolates translated into a decreased UFA/SFA ratio across all three HMF-adapted isolates, which is in contrast with our observations in the furfural-adapted isolates. A decreased UFA/SFA ratio was also reported in freeze-drying stress tolerant *Leuconostoc mesenteroides* obtained via ALE (Kim et al. 2024).

Next, we were interested in knowing how furans affect the fatty acid composition of WT and adapted isolates (Fig. 5). For this purpose, membrane fatty acid composition was analyzed after growing the isolates in the presence of respective inhibitors. When challenged with furfural, a decrease in palmitic acid and an increase in palmitoleic, oleic, and dihydrosterculic acids were observed in the WT. Although changes in the same fatty acids were found in the same direction in the furfural-adapted isolates upon furfural exposure, the extent of the changes was lower or non-significant. This observation suggests that furfural adaptation may induce a form of "homeoviscous adaptation", potentially reprogramming bacterial physiology and baseline membrane composition to maintain optimal fluidity (Sinensky 1974). Because the adapted isolates have already achieved a resilient membrane profile, they do not respond as drastically to acute furfural stress. In contrast, HMF exposure did not affect the fatty acid levels to the same extent as furfural did. In response to HMF, the WT showed lower levels of vaccenic acid and only one adapted isolate showed a significant decrease in palmitic acid. The lower fatty acid modulation in the adapted strains suggests that the long-term adaptation may have reduced the need of acute fatty acid regulation under furan stress.

### Morphological alterations during furan exposure and adaptation

**Fig. 6:**
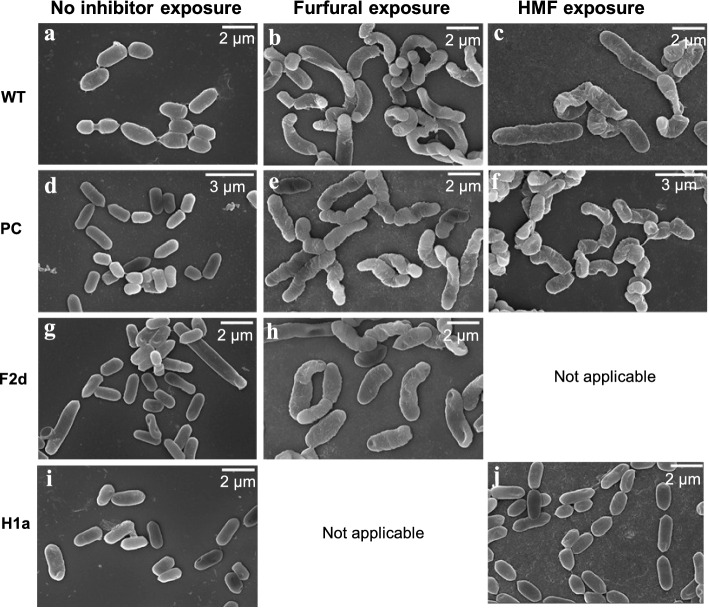
Field emission scanning electron microscopy (FESEM) images of *L. plantarum* JRG2 including wild-type (WT), passage control (PC), furfural- adapted isolate (F2d), and HMF- adapted isolate (H1a). Panels **a**, **d**, **g**, **i** show cells without inhibitor exposure, **b**, **e**, **h** show cell exposed to furfural, and **c**, **f**, **j** show cell exposed to HMF

We examined the effect of furan inhibitors on the cell morphology of WT, PC, and adapted isolates (Fig. 6). When grown without inhibitor exposure, all four exhibited a typical rod-shaped morphology with lengths of 2–3 µm and widths of 700–900 nm. Upon exposure to the inhibitor, however, distinct alterations were observed in the WT and PC isolates. WT cells displayed obvious morphological disruption, with rod-shaped cells becoming curved, flattened, pitted and slightly elongated. Similarly, PC cells showed irregular surfaces and noticeable swelling. Although furfural-adapted isolates also exhibited visibly mild curvature and flattening, the extents of roughness and distortion were less severe than WT or PC.

Similarly, while HMF had a severe morphological impact on WT and PC, the HMF-adapted isolates largely remained unaffected. These observations align very well with parallel non-synonymous mutations observed in the cell-surface related proteins in furfural and HMF-adapted isolates as detailed above. These mutations may cause alterations in the activities of these proteins, potentially leading to changes in cell morphology. Such a hypothesis agrees with an earlier report on mutation in a sortase leading to aberrant envelope physiology in *Streptococcus pyogenes* (Raz et al. 2015) Changes in the cell morphology has also been reported in *E. coli* adapted to sabinene (Wu et al. 2020).

### Improved performance in lignocellulosic hydrolysate

**Fig. 7:**
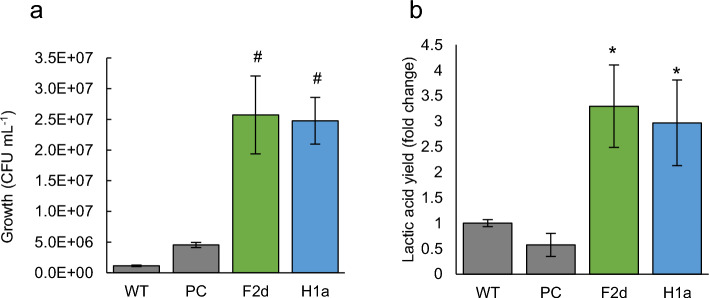
Growth and performance of *L. plantarum* JGR2 in RSH medium. **a** Growth and **b** lactic acid production by WT, PC, furfural-adapted isolate (F2d), and HMF-adapted isolate (H1a) in RSH reported as fold change relative to WT. Statistical analyses were performed by Kruskal–Wallis test followed by post-hoc Dunn’s test, *p* ≤ 0.05; *indicates significant difference from PC, ^#^indicates significant difference from WT

We checked the growth, and lactic acid production ability of the WT, PC and adapted isolates in RSH medium. The CFU data indicates higher growth of adapted isolates in RSH medium as compared to both the WT and PC (Fig. 7a), although it is significantly higher than only WT. Additionally, the adapted isolates produced higher levels of lactic acid than the PC (Fig. 7b). Specifically, the WT, and PC produced 4.62 ± 0.3 µg/mL and 2.64 ± 1.04 µg/mL of lactic acid, respectively, under our experimental conditions. In contrast, the furfural and HMF-adapted isolates produced 3.3- and three- times higher amounts of lactic acid, respectively, compared to the WT (Fig. 4). Although we did not use the classical methanolic extraction and HPLC methods for absolute quantification of lactate production (Zhu et al. 2022), the identical extraction and analytical procedures were applied to all strains. Therefore, our data clearly demonstrate lactate overproduction by the adapted stains. The enhanced lactic acid production in the adapted isolates is likely associated with their improved growth in RSH medium together with their enhanced ability to detoxify furfural.

ALE has been previously used in *S. cerevisiae* to enhance lactic acid production in LCH (Zhu et al. 2022). In *P. putida,* ALE to acetic acid, which is another lignocellulosic growth inhibitor, improved growth and metabolite production in LCH (Zou et al. 2022). As expected through the tolerance development in this study, the adapted isolates showed higher growth and lactic acid production in the hydrolysate than the WT, and PC. Although the absolute lactic acid titer achieved in this study remains below industrially relevant levels, the more than 3-fold increase in its levels observed in the adapted strains demonstrates that adaptive laboratory evolution can improve *L. plantarum’s* performance in rice straw hydrolysate. These results therefore establish a proof of concept for the application of ALE toward the development of microbial strains with enhanced lactic acid production from LCH.

## Conclusion

In this study, ALE was used to successfully generate *L. plantarum* strains with robust, cross-protective tolerance against the predominant lignocellulosic inhibitors, furfural and HMF. We utilized an integrated multi-omics and physiological approach to propose comprehensive mechanistic hypotheses for this adaptation. The genomic identification of parallel non-conservative mutations (for example, in DNA repair and cell envelope functions) strongly correlated with both the global transcriptomic shift and the physically resilient cell morphology observed under furan stress. Furthermore, transcriptomic shifts towards specific detoxification pathways were phenotypically validated by the strains' enhanced biochemical reduction of furfural to furfuryl alcohol, alongside critical baseline modulations in membrane fluidity (altered UFA/SFA and CFA ratios).

While certain correlations of mutations and differential gene expression with phenotypes such as furfural detoxification and cellular structure provide robust evidence of adaptive cellular reprogramming, targeted reverse engineering of these promising genetic candidates remains a logical next step to prove their causative roles in furan tolerance. Since the adapted strains displayed enhanced survival and three-times higher overall lactic acid production in lignocellulosic hydrolysate, future studies focusing on bioprocess optimization for lactic acid production, as well as evaluating cross-tolerance against other pre-treatment byproducts like phenolic compounds, will be essential to harness the industrial bioprocessing potential of these evolved strains. E-supplementary data of this work can be found in the online version of this paper.

## Supplementary Information

Supplementary Material1

Supplementary Material2

## Data Availability

All data generated or analysed during this study are included in this published article [and its supplementary information files] and the raw reads generated through whole genome sequencing and RNA-seq have been deposited in NCBI under BioProject accessions PRJNA1458978 and PRJNA1458965 respectively.
